# Multiple Myeloma: Clinical Updates from the American Society of Clinical Oncology Annual Scientific Symposium 2020

**DOI:** 10.3390/jcm9113626

**Published:** 2020-11-11

**Authors:** Srinivas Devarakonda, Francesca Cottini, Naresh Bumma, Abdullah Khan, Nidhi Sharma, Maria Chaudhry, Don Benson, Ashley Rosko, Yvonne Efebera

**Affiliations:** Division of Hematology, Department of Internal Medicine, The Ohio State University Comprehensive Cancer Center, Columbus, OH 43210, USA; francesca.cottini@osumc.edu (F.C.); naresh.bumma@osumc.edu (N.B.); Abdullah.Khan@osumc.edu (A.K.); nidhi.sharma@osumc.edu (N.S.); maria.chaudhry@osumc.edu (M.C.); Don.benson@osumc.edu (D.B.); Ashley.Rosko@osumc.edu (A.R.); yvonne.efebera@osumc.edu (Y.E.)

**Keywords:** multiple myeloma, stem cell transplant, novel therapies, CAR-T, immunoconjugates

## Abstract

The novel clinical data for plasma cell neoplasms (smoldering myeloma, multiple myeloma, and AL amyloidosis) that were presented in the 2020 American Society of Clinical Oncology virtual scientific symposium are summarized here. Data from large phase-3 studies (CASSIOPEIA, ENDURANCE, and TOURMALINE-MM4 trials) and phase-2 studies (SWOG 1211, GMMG CONCEPT trials) for newly diagnosed multiple myeloma patients who are eligible for autologous stem cell transplantation are described. Updates from previous important studies for multiple myeloma (STaMINA) along with studies on three different chimeric antigen receptor (CAR-) T cell products are also described. Results of clinical studies involving the use of anti-myeloma drugs with novel mechanisms of action such as immunoconjugates, selinexor, venetoclax, monoclonal antibodies, and data on minimal residual disease (MRD) are discussed. These data provide an overview of the efficacy and safety of the various treatments in multiple myeloma and could lead to changes in our clinical practice, which could pave the path for a “cure” in myeloma.

## 1. Introduction

Several important studies in multiple myeloma were presented during this year’s virtual scientific program of the American Society of Clinical Oncology (ASCO). In this report, emerging data involving treatment of newly diagnosed multiple myeloma (MM), relapsed/refractory multiple myeloma, systemic AL amyloidosis, and minimal residual disease are summarized. 

## 2. Newly Diagnosed Multiple Myeloma

### 2.1. CASSIOPEIA trial (Abstract 8538)

CASSIOPEIA study is a randomized phase-3 trial comparing the combination of daratumumab with velcade, thalidomide, and dexamethasone (D-VTd) with VTd alone in patients with transplant-eligible newly diagnosed multiple myeloma (NDMM). Patients were randomized in 1:1 fashion to receive four cycles of induction and two cycles of post-transplant consolidation with D-VTd or VTd alone. In part 1 of the study, D-VTd yielded significantly better response rates including minimal residual disease (MRD) negativity (10^−5^ sensitivity threshold) and reduced the risk of progression or death by 53% compared to VTd alone [1]. At the ASCO annual symposium this year, a subgroup analysis of this study based on baseline slimCRAB criteria of the patients was presented [2]. In 2014, the International Myeloma Working Group (IMWG) revised the diagnostic criteria for multiple myeloma (MM), adding three validated biomarkers to the conventional CRAB (elevated Calcium, Renal failure, Anemia, Bone disease) in an attempt to diagnose MM early, thereby preventing end organ damage [3]. They were ≥60% clonal marrow plasma cells, serum free light-chain ratio ≥100, and more than one focal lesion on magnetic resonance imaging (MRI)—making slimCRAB criteria. Of the 1085 patients in this study, 81 patients had MM diagnosed by the slim criteria without CRAB features. Fewer patients had advanced stage (4% vs. 16% stage III) and high-risk (HR) features (11% vs. 16%) in the slim-only group with better performance status (22% vs. 54% ECOG ≥ 1) compared to the CRAB group. All the response rates to D-VTd and VTd including overall response rate (ORR), stringent complete response (sCR), complete response (CR), and MRD negativity were similar between the slim-only and CRAB groups. After a median follow-up of 18.8 months, progression-free survival (PFS) was not significantly different between the two groups. Even in the slim-only group, D-VTd was superior to VTd alone in terms of all the responses, including MRD negativity.

Discussion: D-VTd seems to be efficacious and superior to VTd even in slim-only MM patients. The deeper responses seen with D-VTd in the slim-only group might translate into PFS and possibly overall survival (OS) benefit with longer follow-up. Early diagnosis probably accounts for fewer patients with advanced stage MM and poor performance status in the slim-only group, although the small number of patients limits definitive conclusion.

Despite the improvement in the outcomes of patients with multiple myeloma with the therapeutic advances, patients with high-risk multiple myeloma (HR MM) continue to have poor prognosis [4]. Several trials are being conducted investigating novel induction regimens to achieve better outcomes for this group of patients. A few of those trials presented at the recent ASCO virtual scientific symposium are discussed here.

### 2.2. GMMG CONCEPT Trial (Abstract 8508)

**Figure 1:**
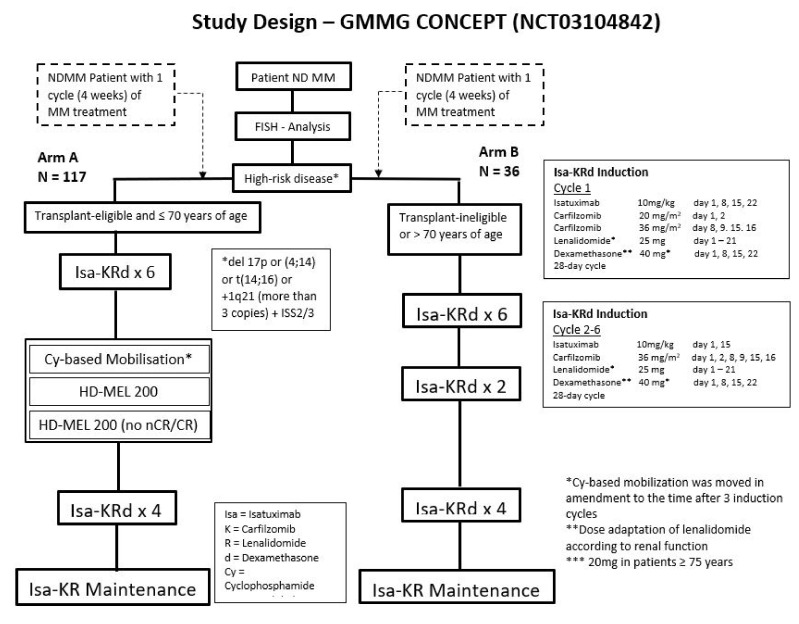
Study design and induction regimen of the GMMG CONCEPT trial.

The results of the pre-specified end of induction interim analysis (IA) of the GMMG CONCEPT trial investigating the role of the quadruplet regimen isatuximab plus carfilzomib, lenalidomide, and dexamethasone (Isa-KRD) in high-risk, newly diagnosed multiple myeloma (HR NDMM) were presented [5]. This is a phase-2, multi-center, open-label study. HR MM was defined by International Staging System (ISS) stage 2 or 3 with the presence of del17p, or t(4;14), or t(14;16), or >3 copies 1q21. In the trial, patients received six cycles of Isa-KRd induction, four cycles of Isa-KRd consolidation, and Isa-KR maintenance until progressive disease or toxicity. Transplant-eligible patients (arm A) received high-dose therapy followed by stem-cell rescue, while transplant-ineligible patients (arm B) received two additional cycles of Isa-KRd induction. Study design and dosage of the drugs in the induction regimen is presented in Figure 1. The primary endpoint is MRD negativity measured by next-generation flow after consolidation. In this IA, overall response rates (ORR) after induction were reported. Of the total accrual goal of 153 patients, 50 patients were included in the IA report for ORR. Of the 50 patients, 46 were from arm A and 4 patients were from arm B. In arm A, 39/46 patients and in arm B 4/4 patients completed induction treatment. ORR was 100%, with 90% of the patients achieving very good partial response (VGPR) or better. In arm A, 20/33 patients became MRD negative during induction. Grade 3/4 treatment-emergent adverse events (≥10%) with Isa-KRd included neutropenia, leukopenia, and thrombocytopenia. Main non-hematologic toxicities grade 3/4 were hypertension and infection. Median stem-cell yield was 6.6 × 10^6^CD34 + cells/kg.

Discussion: The GMMG CONCEPT trial is one of the first studies that included only NDMM patients with high-risk disease. The most common high-risk feature in this group was deletion 17p. Isa-KRD seems to be an efficacious regimen in HR NDMM with ORR and depth of response (VGPR or better) at the end of induction (100%/90%) comparable to other four-drug regimens such as Dara-VRD (98%/>70%) [6] or Dara-KRd (100%/92%) [7]. More importantly, the high response rates seen in the CONCEPT trial are in an exclusive HR MM population unlike these other trials that included patients with both standard and high-risk disease. Incidence of grade 3/4 hematological toxicity seems comparable to other studies using four-drug regimens for induction therapy of NDMM (neutropenia/thrombocytopenia = 32%/16% in GRIFFIN study using Dara-VRD). At 24 months in the GRIFFIN study, rates of progression-free survival (PFS) and overall survival (OS) were greater than 85% and 90% respectively in all the included patients (high-risk and standard risk patients); no PFS or OS are available for the CONCEPT trial yet.

### 2.3. SWOG-1211 Trial (Abstract 8507)

Primary analysis of SWOG-1211, a randomized phase-2 trial of bortezomib, lenalidomide, and dexamethasone with/without elotuzumab for high-risk, newly diagnosed multiple myeloma (HR NDMM) was presented [8]. The trial design includes comparing eight cycles of lenalidomide, bortezomib, and dexamethasone (RVd) induction followed by dose-attenuated RVd maintenance until disease progression with or without elotuzumab (Elo) in a 1:1 randomization. RVd and Elo were given at standard doses and schedule during induction. During maintenance, lenalidomide was given at a dose of 15 mg PO (by mouth) on days 1−21, bortezomib 1.0 mg/m^2^ subcutaneous (SC) on days 1, 8, 15, and dexamethasone 12 mg PO on days 1, 8, 15 along with Elo 10 mg/kg IV on days 1, 15 of a 28-day cycle. Stem-cell collection was allowed after cycle 2 on protocol but the actual high dose therapy with stem cell rescue was deferred until progression/relapse off-protocol. High-risk MM was defined by one of the following: gene expression profiling high-risk (GEP^hi^), t(14; 16), t(14; 20), del(17p) or amplification 1q21, primary plasma cell leukemia (pPCL), and elevated serum LDH (>2X ULN). Median progression-free survival (PFS) was the primary end-point and secondary endpoints included overall response rate (ORR), safety, and overall survival (OS). Of the 100 patients enrolled, 52 received RVd and 48 received Elo-RVd. One-third of the patients had International Staging System (ISS) III. 17p deletion was present in 42% in the RVd arm and 31% in the RVd-Elo arm. Eight percent of the patients in the RVd arm and 6% in the RVd-Elo arm had pPCL. With a median follow-up of 53 months (mos.), no difference in median PFS was observed [RVd-Elo = 31 mos., RVd = 34 mos., HR = 0.968 (80% CI = 0.697–1.344), *p* = 0.449]. No difference in OS was observed (RVd-Elo = 68 mos, RVd = not reached, HR = 1.279 (80% CI: 0.819, 2.000), *p*-value = 0.478). No differences in the safety profile were observed between the groups except for more grade 3 or higher adverse events (AE) such as neutropenia, infections, and sensory neuropathy seen with the addition of Elo. 

Discussion: The addition of elotuzumab to RVd induction and maintenance did not improve patient outcomes. This is the first randomized study to be conducted exclusively in HR NDMM and has incorporated a wide range of high-risk features including pPCL, and 1q21 amplification and t(14;20). However, it has to be noted that high-dose therapy with stem-cell rescue, which is a commonly utilized treatment for the management of HR MM, was not performed in the upfront setting in this study. Although prior studies of Elo/dex with pomalidomide [9] and lenalidomide [10] separately in relapsed or refractory MM (RRMM) showed promising results, the combination of RVd with Elo induction and maintenance did not fare better than RVd alone in high-risk NDMM. In fact, the benefit from Elo was greater when added to pomalidomide than lenalidomide and it could be just due to the superior efficacy of pomalidomide as a second-generation immunomodulator. 

### 2.4. Endurance (E1A11) Trial (Abstract LBA3)

The results of the large phase-3 ENDURANCE (E1A11) trial comparing carfilzomib, lenalidomide, and dexamethasone (KRd) versus bortezomib, lenalidomide, and dexamethasone (VRd) for initial therapy of newly diagnosed multiple myeloma (NDMM) were reported in the “Late-Breaking Abstracts” session [11]. The superiority of carfilzomib over bortezomib in the relapsed/refractory myeloma setting was shown earlier in the phase-3 ENDEAVOR trial [12]. The ENDURANCE trial aimed to examine if KRd improves PFS compared to VRd in NDMM (current results), and whether indefinite maintenance with lenalidomide improves OS compared with two-year maintenance (to be analyzed once data matures). Patients with NDMM were randomized to receive VRd or KRd in a 1:1 fashion for 36 weeks followed by a second randomization (1:1) to indefinite versus two years of lenalidomide maintenance. Patients with high-risk features (del17p, t (14;16), t(14;20), plasma cell leukemia, or high-risk GEP70 profile) were excluded. Arm A included induction with 12 cycles of VRd, while arm B received 9 cycles of KRd induction at standard doses and schedules. Maintenance included lenalidomide 15 mg d 1–21 every 4 weeks for 24 cycles in arm C and until progression or excessive toxicity in arm D. Stem-cell collection was allowed after 12 weeks of therapy at the discretion of the investigator. The first co-primary endpoint was PFS for the induction randomization, and the second co-primary endpoint was OS for the second randomization. The study accrued 1087 patients (VRd = 542, KRd = 545). The median age of patients was 65 years. Treatment, efficacy, and toxicity data are shown in Table 1. At the second of three planned interim analyses, with PFS HR = 1.04 (95% CI, 0.8 to 1.3, *p* = 0.74), KRd was not superior to VRd. VRd yielded a median PFS of 34.4 months while it was 34.6 months with KRd. No differences were seen based on age (<65 or ≥65), presence or absence of t(4;14), or ISS stage. The three-year OS (95% CI) was similar with VRd 84% (80 to 88) and KRd 86% (82 to 89). A significantly higher rate of cardio–pulmonary and renal toxicity was observed with KRd, while neuropathy rates were higher with VRd.

Discussion: One of the key features of this trial is that it is a head-to-head comparison of two triplet regimens for upfront treatment of NDMM in contrast to triplet vs. doublet, where triplet often performs better in comparison with doublet. Patients with high-risk multiple myeloma, except those with t(4;14) or those who were planned to undergo early autologous stem cell transplant (auto-SCT) were not included in this study. Exclusion of high-risk myeloma might have accounted for the lack of superiority of carfilzomib over bortezomib. It is of note that adherence to treatment was poor in this trial, with 38% in the KRd arm and 57% in the VRd arm failing to complete the planned number of cycles of induction therapy. In terms of efficacy, KRd was superior to VRd (VGPR or better 73.8% versus 64.7%, *p* = 0.002). The number of patients who proceeded to auto-SCT was comparable between the two groups, which makes both regimens suitable for induction in transplant-eligible patients. The median PFS achieved with VRd in this trial (34.4 months) is much lower compared to that seen in the SWOG0777 trial (43.3. months) [13]. This could probably be related to the failure to complete induction therapy as planned (57% patients in the ENDURANCE trial vs. 43% in the SWOG0777 trial failed to complete planned induction therapy). Also, longer follow-up might lead to improvement in the PFS with VRd in the ENDURANCE trial. This study provides the rationale for the use of carfilzomib for induction in patients with significant underlying peripheral neuropathy and alternatively bortezomib in patients with significant baseline renal, cardiac, or pulmonary disease. VRd remains the standard induction therapy for non-high-risk myeloma and the backbone for building upon with the addition of monoclonal antibodies and other drugs.

### 2.5. BMT CTN 0702 (STaMINA) Trial (Abstract 8506)

The initial results of the BMT CTN 0702 (STaMINA) trial investigating post auto-SCT strategies in the upfront treatment of MM were reported by Stadtmauer et al. in 2018 [14]. During this year’s ASCO virtual scientific symposium, the six-year follow up for STaMINA and the results of lenalidomide (Len) discontinuation beyond three years were presented [15]. Using intent-to-treat (ITT) analysis, six-year PFS and OS were the same among Auto/Auto (43.9%; 73.1%), Auto/RVD (39.7%, 74.9%), and Auto/Len (40.9%, 76.4%) (*p* = 0.6; *p* = 0.8). Protocol defined high-risk disease, (HR = 1.53, *p* < 0.0001) and age (*p* = 0.03) were adverse risks for PFS. In as-treated analysis, six-year PFS was 49.4%, 39.7%, and 38.6% for Auto/Auto, Auto/RVD, and Auto/Len respectively (*p* = 0.015). Six-year PFS in as-treated analysis for high-risk patients were 43.7% and 32% for Auto/Auto and Auto/Len, respectively (*p* = 0.03). Landmark analysis at 38 months showed that patients who discontinued Len maintenance after 38 months had inferior PFS (61% vs. 79.5% at 6 y; HR = 1.91, *p* = 0.0004) but similar OS compared with those who continued it until progression. Incidence of all second primary malignancies (SPM) (84 cases with 45 hematological malignancies) was associated with age and was no different among the three arms. 

Discussion: In this large study comparing post auto-SCT strategies, tandem auto-SCT or post auto-SCT consolidation did not improve outcomes but as-treated analysis suggested a PFS benefit for tandem auto-SCT, driven mainly by patients with high-risk MM. Although Auto/Len remains the standard of care for patients with NDMM, given the PFS benefit with tandem auto-SCT it could be considered for young and fit patients with high-risk disease. Len discontinuation even after three years was associated with inferior PFS. While there is no consensus on the duration of maintenance therapy in NDMM yet, this study provides rationale to continue maintenance therapy beyond three years in the absence of progression or excessive toxicity.

### 2.6. TOURMALINE-MM4 Trial (Abstract 8527)

The results of the large phase-3, randomized TOURMALINE-MM4 trial comparing the oral proteasome inhibitor ixazomib vs. placebo maintenance for NDMM patients not undergoing auto-SCT were presented [16]. The trial was conducted to explore more options for maintenance therapy given the toxicity and route of administration of other approved options. Patients with transplant-ineligible NDMM were enrolled after receiving 6–12 cycles of any induction therapy and achieving at least a partial response. They were randomized in a 3:2, double-blind fashion to receive either ixazomib (3 mg, cycles 1–4, then, if tolerated, 4 mg, cycle 5 onwards) or placebo on days 1, 8, 15 of a 28 day cycle for a maximum of 2 years. Patients were stratified by prior proteasome inhibitor (PI) exposure (yes vs. no), pre-induction ISS disease stage (I/II vs. III), age (<75 vs. ≥75 years), and post-induction best response (CR/VGPR] vs. PR). Primary endpoint was PFS. Overall median age of patients was 73 years and 38% of patients were aged ≥75 years. Thirty-five percent of patients had ISS stage III and 22%/40%/38% had CR/VGPR/PR post induction. Overall, 82% of patients received a PI and 33% an immunomodulatory drug as part of their induction regimen. Results of the study are shown in Table 2. Common treatment-emergent adverse events (TEAEs) for ixazomib vs. placebo included nausea (27% vs. 8%), vomiting (24% vs. 4%), and diarrhea (23% vs. 12%) and were mostly grade 1–2. There was no adverse impact on the quality of life (QoL) of the patients who received ixazomib. New primary malignancies were seen in 5.2% of patients who received ixazomib compared to 6.2% who received placebo. 

Discussion: Ixazomib, the first oral proteasome inhibitor maintenance therapy in non-transplant eligible NDMM patients, showed a clinically meaningful 34% reduction in the risk of progression or death, with a well-tolerated safety profile. Significant PFS benefit was noted for patients in VGPR or better post induction. Specifically, in this study, median PFS was 17.4 months with ixazomib and 9.4 months with placebo, while median PFS was 26 months (95% CI 22–31) with lenalidomide and 11 months (5–23) with observation (HR 0.44 [95% CI 0.37–0.53]; *p* < 0.0001) in the Myeloma XI study for non-transplant eligible patients [17]. Even though studies to directly compare ixazomib with lenalidomide in the non-transplant maintenance setting are missing, ixazomib is an effective alternative maintenance option in patients who are intolerant to lenalidomide.

## 3. Relapsed or Refractory Multiple Myeloma (RRMM)

Despite the rapid evolution of the treatment landscape of MM over the past decade with the introduction of several drugs such as proteasome inhibitors, immunomodulators, and monoclonal antibodies (against CD38 and SLAMF7), most patients eventually relapse and succumb to the cancer. This highlights the need for the development of treatment strategies with novel mechanism of action that can overcome the tumor resistance to conventional therapies. Chimeric antigen receptor (CAR-) T cell therapy is a promising immunotherapeutic approach resulting in selective cancer cell death and has been shown to have potential in relapsed/refractory acute lymphoblastic leukemia and diffuse large B cell lymphoma [18,19]. CAR-T cell therapy directed against B-cell maturation antigen (BCMA) is being tested in MM with promising activity so far, especially in the relapsed/refractory setting. Impressive results including rapid and deep responses—and including MRD negativity—are being demonstrated even in heavily pre-treated patients who relapsed on all available therapies including monoclonal antibodies. Updates from the trials involving some of the CAR-T products presented at the ASCO symposium are discussed below.

### 3.1. Updates from CAR-T Cell Therapies

#### 3.1.1. CARTITUDE-1 Study (Abstract 8505)

JNJ-4528 is a chimeric antigen receptor (CAR-) T cell therapy being investigated in the phase 1b/2 CARTITUDE-1 study for the treatment of RRMM. It is identical to the CAR construct used in the LEGEND-2 study that was conducted in China and the results were promising, and targets two different epitopes of BCMA [20]. The initial safety and efficacy results of JNJ-4528 were presented at the American Society of Hematology (ASH) symposium in 2019 and it was shown to be safe and effective at a follow up of six months [21]. The investigators presented their findings at a median follow up of nine months at the recent ASCO virtual scientific symposium [22]. Eligibility criteria included at least three prior lines of therapy, refractoriness to PI or immunomodulatory drug (IMiD), and prior exposure to anti-CD38 monoclonal therapy. Thirty patients with measurable disease were enrolled to the study and twenty-nine were treated. The median age of the patients was 60 years and with a good performance status of ECOG 0–1. Median dose of the CAR-T product was 0.72 × 10^6^ cells/kg. All the patients who received JNJ-4528 achieved a response with an impressive 97% VGPR or better and 86% stringent complete response (sCR). Nearly four-fifths of the patients who achieved complete response (CR) were MRD negative, indicating the depth of response this treatment conferred. At a median follow-up of 11.5 months, 22 of the 29 treated patients are alive and progression-free. The nine-month PFS rate was 86% (95% CI, 67–95). Cytokine release syndrome (CRS) was frequent but mostly low grade, with a median time of onset of seven days and median duration of four days. Neurotoxicity was mostly low grade with one grade-3 event. Cytopenias were seen in more than two-thirds of the patients, mostly grade-3 or higher but resolved by two months after treatment. Infectious complications were infrequent and no deaths were reported secondary to infection.

Discussion: The results seen with JNJ-4528 CAR-T therapy are impressive, with an ORR of 100%, 97% VGPR or better, and nearly 80% MRD negativity in patients with CR in a heavily pre-treated population. The number of median prior lines of therapy was five, with 86% having triple refractory and 28% pentarefractory myeloma. One-third of the patients had high-risk cytogenetics. Most of the patients underwent prior autologous stem cell transplantation. It is to be noted that the rate and depth of response were independent of the BCMA expression on myeloma cells at baseline. Longer follow-up would provide data about the durability of the responses achieved. Phase-2 and-3 studies with JNJ-4528 have been initiated and are ongoing.

#### 3.1.2. KarMMa Study (Abstract 8503)

Safety and efficacy of Idecabtagene-vicleucel (ide-cel, bb 2121), a CAR-T product targeting the B-cell maturation antigen (BCMA), has been tested in the phase 2 KarMMa study and the data was presented [23]. Patients with RRMM who received at least three prior lines of therapy including PI, IMiD, and anti-CD38 antibody and refractory to the last prior therapy were eligible for the study. ORR was the primary end point with other responses and quality of life (QOL) being secondary end points. Patients were treated with three ide-cel doses of CAR-T cells.

One hundred and twenty eight patients were treated with ide-cel. The study met its end points of ORR and CR. Results are shown in Table 3. At a median follow up of 13.3 months across all target dose levels, ORR was 73% with 33% CR. More impressively, responses were deep with 26% of the patients in CR and 39% of the patients in VGPR or better achieving MRD negativity (sensitivity <10^−5)^ by next-generation sequencing (NGS). Best responses were seen at a dose of 450 × 10^6^ cells with an ORR of 82% that consisted of 65% with VGPR or better and half of them achieving MRD negativity. Duration of response (DoR) and PFS also increased with the dose of ide-cel, with the best responses seen at the 450 × 10^6^ cells dose. Data for overall survival is immature at the time of presentation. CRS was seen in the majority (84%) of the patients but was mostly grade 1 or 2 and rarely grade 3 even at higher dose levels. It was short-lived and resolved with the use of Tocilizumab or corticosteroids. Neurotoxicity occurred infrequently, was mostly low grade and resolved spontaneously without intervention. There were no grade 4 or 5 events. Cytopenias were very common, mostly grade 3 or higher and unrelated to the dose, probably related to the poor bone marrow reserve from extensive prior treatment and/or tumor burden. Delayed cell recovery was seen in many patients. Infections were very common at all dose levels. Five patients died within two months of treatment with ide-cel, of which three were treatment related.

Discussion: Ide-cel or bb2121 is another CAR-T product effective in the management of heavily pre-treated RRMM. The patient population was heavily pre-treated and nearly all patients had prior autologous stem-cell transplants, with one-third of them having received more than one transplant. Most patients were triple-refractory (PI, IMiD, and anti-CD 38) and 94% were refractory to anti-CD38 antibody. In spite of that, the median duration of response (DOR) and PFS were 11.3 months and 12.1 months respectively at the highest dose of CAR-T cells, superior to those conferred by other regimens used in the treatment of RRMM. In addition, ide-cel was efficacious irrespective of age, cytogenetic risk profile, tumor burden, BCMA expression, and presence of extramedullary disease. Efficacy was seen even in the triple and penta-refractory patients and those who received bridging therapy without response, making it an attractive salvage treatment option for this hard-to-treat population. The addition of prophylactic antibiotic, antifungal, and antiviral agents may help reduce infection.

#### 3.1.3. EVOLVE Study (Abstract 8504)

Results of the phase 1/2 EVOLVE study using orva-cel at doses of 300, 450, and 600 × 10^6^ cells were presented [24]. Orva-cel is an investigational BCMA-directed CAR-T cell product with a fully human binder. The results of this product at lower doses of 50 and 150 × 10^6^ cells were presented at the American Society of Hematology annual meeting in 2018. Current report includes the safety and efficacy outcomes of patients who were treated with the higher doses of orva-cel.

A total of 62 patients with RRMM who received three or more lines of therapy including a proteasome inhibitor (PI), immunomodulatory drug (IMiD), and anti CD-38 antibody were treated with orva-cel at 300, 450, and 600 × 10^6^ CAR-T cells after lymphodepletion with fludarabine and cyclophosphamide. The median age of patients was 61 years. Median time from diagnosis was 7 years and the patients were heavily pre-treated with a median of six prior regimens. Most of the patients were penta-exposed, including two PIs, two IMiDs, and anti-CD-38 antibody, and had received prior auto-SCT. Two-thirds of the patients received bridging therapy and most of them were refractory to it. Results are shown in Table 4. At a median follow-up of 6.9 months, ORR and VGPR or better were 92% and 68% across all doses respectively and PFS was not reached. MRD negativity by NGS (10^−5^ sensitivity) was observed in 84% of the patients across all dose levels at three months. Cytopenias were common but were quick to improve. Grade ≥3 infections occurred in 13% of the patients. One patient died from grade 5 macrophage activation syndrome (MAS) at the 450 × 10^6^ cells dose level. Enrollment is ongoing for the phase-2 study with a dose level of 600 × 10^6^ cells.

Discussion: Orva-cel has a fully human binder with low affinity for soluble BCMA and is active on target cells with low BCMA density with a spacer to increase the binding. The initial results using higher doses of orva-cel seem promising with impressive overall response rates in a heavily pre-treated population. Two-thirds of the patients achieved VGPR or better with MRD negativity in 84% of patients achieving PR or better. Testing for MRD negativity was done in patients achieving PR or better in this study, while other studies used VGPR or better as the threshold for testing MRD status—in fact, according to the IMWG MRD criteria, CR is needed for MRD testing [25]. Macrophage activation syndrome (MAS) or hemophagocytic lymphohistiocytosis (HLH) was a unique adverse event (AE) observed with orva-cel that led to the death of one patient at the 450 × 10^6^ cells dose level. MAS/HLH is likely from the exaggerated immune response incited by this particular CAR-T construct and further research is needed to fully understand the risk factors for developing this AE and ways to prevent it. Although higher-grade cytopenias were more common in patients treated with the higher CAR-T cell doses, they resolved within two months. The results for the higher dose levels are early, and longer follow-up is needed to determine the durability of response and survival outcomes such as PFS and OS.

A comparative analysis of the above three CAR-T products is presented in Table 5.

### 3.2. Immunoconjugates

Immunoconjugates are a novel class of drugs that are being tested in the treatment of RRMM. Belantamab mafodotin (GSK2857916) is a first-in-class, anti-BCMA immunoconjugate with an afucosylated, humanized IgG1 anti-BCMA monoclonal antibody conjugated by a protease-resistant maleimidocaproyl linker to a microtubule-disrupting agent, monomethyl auristatin F (MMAF) [26]. Belantamab mafodotin binds to BCMA and kills multiple myeloma cells via a multimodal mechanism, including delivery of MMAF to BCMA-expressing multiple myeloma cells, thereby inducing apoptosis; enhancing antibody-dependent cellular cytotoxicity and antibody-dependent cellular phagocytosis; and inducing immunogenic cell death [27].

#### DREAMM-6 Study (Abstract 8502)

DREAMM-6 is an ongoing, two-part, two-arm study evaluating the safety, tolerability, and clinical activity of belantamab mafodotin (bela maf) in combination with lenalidomide/dexamethasone(Rd)–BRd (arm A) or bortezomib/dexamethasone (Vd)–BVd (arm B) in patients previously treated with ≥1 prior therapy. Safety and tolerability of the 2.5 mg/kg single dose cohort BVd (arm B) in patients with RRMM was reported [28].

Part 1 of the study consisted of dose escalation at 2 single doses of bela maf (2.5 mg/kg and 3.4 mg/kg) given on day 1 along with bortezomib SC 1.3 mg/m^2^ on d 1, 4, 8, 11 and dexamethasone 20 mg on d 1,2, 4, 5, 8, 9, 11 and 12 of a 21-day cycle. It had six patients enrolled to each dose level. Part 2 consisted of dose expansion of bela maf at 2 doses (2.5 and 3.4 mg/kg) given as single/split in combination with bortezomib and dexamethasone at the same doses as in dose escalation. Bela maf 2.5 mg/kg split is given as 1.25 mg/kg on days 1 and 8, whereas 3.4 mg/kg split is given as 1.7 mg/kg on days 1 and 8 of a 21-day cycle. Results of the 2.5 mg/kg single dose cohort of the BVd are presented in this report. Safety, tolerability, and ORR of the BVd combination were the primary end points, while preliminary clinical activity was one of the secondary end points. At the time of data submission, 18 patients received a median of 18.2 weeks of treatment with the 2.5 mg/kg single dose bela maf with Vd (6 in part 1 and 12 in part 2). No dose-limiting toxicities were observed in part 1 at the time of reporting. Corneal events (including keratopathy, blurred vision, and dry eye) and thrombocytopenia were the most frequently reported adverse events (AE). There were no grade-5 events and no grade-4 corneal events. There were no AEs leading to discontinuation of bela maf. 

Discussion: Preliminary data from this study shows acceptable safety profile for the combination of belantamab with bortezomib/dexamethasone. With bela maf at 2.5 mg/kg (*n* = 18), overall response rate (ORR) was 78% (95% CI, 52.4–93.6) with 50% obtaining a VGPR. The duration of response is not yet reached. It is of note that bortezomib refractory patients were not excluded from the study and the ORR with the BVd combination is greater than the ORR with Vd in RRMM with >1 prior line of therapy [29,30,31].

### 3.3. Novel Drugs

Selinexor is an Oral, Selective Inhibitor of XPO1-Mediated Nuclear Export, leading to the reactivation of tumor suppressor proteins. The efficacy of Selinexor with low dose dexamethasone in triple-class refractory RRMM was shown in the STORM study, where the ORR was 26.2% and led to the approval by the FDA for this indication [32].

#### 3.3.1. BOSTON Study (Abstract 8501)

**Figure 2:**
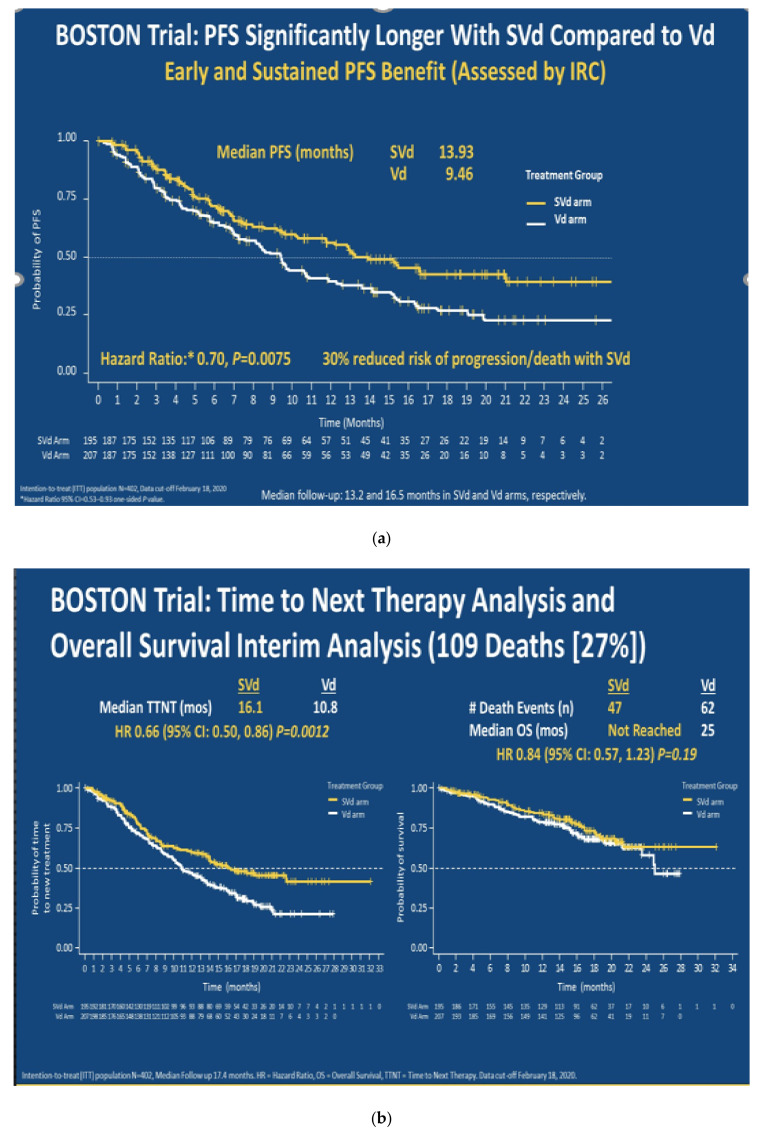
Outcomes of the BOSTON study. (**a**) Progression-free survival analysis (**b**) Time to next therapy and overall survival interim analysis.

The results of the phase-3, randomized BOSTON study comparing once-weekly Selinexor in combination with bortezomib and dexamethasone (SVd) to twice weekly bortezomib/dexamethasone (Vd) in patients with RRMM were reported [33]. The efficacy and tolerability of once-weekly Selinexor in combination with bortezomib and dexamethasone in RRMM was shown in a phase 1b/2 study earlier and it was effective in both proteasome inhibitor sensitive and refractory disease) [34]. In the BOSTON study, PFS was the primary end point with ORR, OS, and peripheral neuropathy being the secondary end points. Randomization was stratified by treatment with prior PI therapies, number of prior anti-MM regimens (1 vs. >1), and Revised International Staging System (R-ISS; Stage III vs. I or II). Upon progression, patients on Vd could cross over to either SVd if they were able to tolerate continued bortezomib, or selinexor and dexamethasone (Sd) if they developed intolerance to bortezomib. Of the 402 patients that were enrolled, 195 received SVd (S at 100 mg PO days 1,8,15,22,29; V at 1.3 mg/m2 SC days 1,8,15,22; and dex 20 mg PO days 1,2,8,9,15,16,22,23,29,30) of a 35-day cycle and 207 received Vd (V 1.3 mg/m2 SC days 1,4,8,11; dex 20 mg PO days 1,2,4,5,8,9,11,22) of a 21-day cycle (cycles 1–8). After cycle 9, Vd was given weekly in 35-day cycles. Patients in both arms continued treatment until progression or toxicity. SVd was associated with a significantly higher ORR than Vd (76.4% vs. 62.3%, *p* = 0.0012). SVd significantly prolonged PFS vs. Vd (median 13.93 vs. 9.46 months, HR = 0.70, *p* = 0.0066). Median OS was not reached on SVd vs. 25 months on Vd (*p* = 0.28) (Figure 2). Most frequent treatment-related adverse events (TRAE) (grade ≥3) for SVd vs. Vd were thrombocytopenia (35.9% vs. 15.2%), fatigue (11.3% vs. 0.5%), and nausea (7.7% vs. 0%). Peripheral neuropathy (PN) rates (grade ≥2) were significantly lower with SVd that included weekly bortezomib vs. Vd in which bortezomib was given twice weekly (21.0% vs. 34.3%, *p* = 0.0013). Disease progression was the most common reason for treatment discontinuation and occurred more often in the Vd arm (52% vs. 34%), followed by AE/toxicity (11% in Vd arm vs. 17% in SVd arm). 

Discussion: Selinexor given once weekly with bortezomib and dexamethasone seems tolerable and prolongs PFS compared to the doublet of PI and steroid in RRMM. Median duration of study treatment and discontinuation of treatment due to AE/toxicity with SVd are comparable to those seen with other triplet regimens with Vd backbone in patients with RRMM who have received two or more prior lines of therapy [29,30,31]. It is to be noted that 50% of the patients in the SVd arm had high-risk cytogenetics including del 17p, t(4;14), t(14;16), or amp1q21, while high-risk features were seen in 15–22% of the patients in the treatment arm in other trials involving PI-based triplet regimens for RRMM [29,30,31,35]. Another unique feature of this trial was that it investigated a once-weekly velcade dosing to a twice-weekly control arm.

#### 3.3.2. STOMP Study (Abstract 8530)

Once-weekly selinexor, kyprolis (carfilzomib), and dexamethasone (SKd) is being tested in RRMM in the STOMP study and initial results were presented [36]. This is a phase 1b/2 dose escalation/dose expansion study evaluating various doses of selinexor and kyprolis in kyprolis-naïve patients with RRMM. Oral selinexor was dosed once-weekly (QW) at 80 or 100 mg. Carfilzomib was dosed QW (on days 1, 8, and 15 of a 28-day cycle) at 56 mg/m^2^ or 70 mg/m^2^. Dexamethasone PO was dosed at 40 mg QW. The primary end points of the study are maximum tolerated dose (MTD), recommended phase-2 dose (RP2D), efficacy, and safety of SKd. At the time of reporting, 24 patients were enrolled. Their median age was 71 years. The median number of prior regimens was three. The maximum tolerated dose (MTD) was selinexor 80 mg QW, carfilzomib 56 mg/m^2^ QW, and dexamethasone 40 mg QW. The ORR was 70.8% with 50% VGPR or better. With a median follow-up period of 4.7 (1.8–16.3) months, median PFS has not been reached. Common treatment-related adverse events (TRAE) were thrombocytopenia, nausea, anemia, fatigue, anorexia, weight loss and neutropenia. Grade-3 or higher TRAE included thrombocytopenia, anemia, neutropenia, fatigue, anorexia, vomiting, and hyponatremia. 

Discussion: The combination of SKd seems to be efficacious in this small cohort with RRMM (VGPR or better in half the patients) and with no new safety signals at a short median follow-up period. Longer follow-up and phase-3 study will shed light on the continued efficacy and safety of this combination. It is of note that more half the patients had prior exposure to daratumumab and all of them were bortezomib exposed with half of them refractory to it. The investigators consider the side effects to be a function of dose and schedule and that they can be alleviated with dose modification and supportive care. Selinexor is being tested with several other drugs for this indication as part of the STOMP study. 

#### 3.3.3. TAK-079 (Abstract 8539)

TAK-079 is a cytolytic monoclonal anti-CD38 antibody that depletes target cells by multiple mechanisms including apoptosis, antibody-dependent cellular cytotoxicity (ADCC), complement-mediated cytotoxicity (CDC), and antibody-dependent cellular phagocytosis (ADCP) [37].

Preliminary results of a phase 1b study of the efficacy and safety of TAK-079 in patients with RRMM were presented [38]. Eligibility criteria included patients having RRMM, who had received three or more prior lines of therapy including PI, IMiD, alkylating agent with or without anti-CD38 antibody, were refractory or intolerant to a PI and IMiD, and having disease progression on the most recent therapy. TAK-079 was given as a SC injection weekly for eight doses, then every other week for eight doses, then monthly until disease progression (PD) or unacceptable toxicity. 

Forty-one patients were enrolled across five fixed-dose cohorts (TAK-079 45-135-300-600-1200 mg SC) as of March 20, 2020. The median age of patients was 67 years. Median number of prior therapies was 3.5. At study entry, 65% were refractory to both an IMiD and PI, 85% refractory to last line of therapy, and 25% of patients were previously exposed to at least one anti-CD38 monoclonal antibody. No infusion-related reactions (IRR) were reported. Drug-related adverse events (AEs), any grade, occurring in at least 10% of patients were fatigue, neutropenia, anemia, and upper respiratory tract infection. Drug-related grade ≥3 AEs were neutropenia, anemia, parainfluenza virus infection and diverticulitis. The only drug-related serious adverse event (SAE) was one occurrence of grade-3 diverticulitis. No drug-related grade-4 AEs, AEs leading to study discontinuation, or on-study deaths were reported. A recommended phase-2 dose (RP2D) of 600 mg was selected. At the RP2D dose, 17 patients were response evaluable and their ORR was 24% with a PFS of 5.8 months. The ORR in anti-CD38 naïve patients was 36% with a PFS of 6.7 months. At a median follow-up of 9.2 months, the median DoR was not reached. At 9 and 12 months, 41% and 31% of the response evaluable patients remained progression-free. 

Discussion: TAK-079 seems to be an efficacious and safe drug in RRMM with outcomes comparable to those reported with other anti-CD38 antibodies in this setting, either naïve or exposed to anti-CD38 therapy (ORR with Daratumumab 33% in SIRIUS trial and 37% (IV arm) in the COLUMBA trial, 26% with Isatuximab) [39,40,41]. The response of one anti-CD38 agent in patients who have been exposed or are refractory to another anti-CD38 agent are yet unknown. This is an area of utmost importance and needs to be studied.

#### 3.3.4. CC-92480 (Abstract 8500)

CC-92480 is a novel cereblon (CRBN) E3 ligase modulator (CELMoD) agent designed for rapid, maximal degradation of Ikaros and Aiolos. In vitro, it showed enhanced antiproliferative and tumoricidal activity in MM cell lines, including those resistant to lenalidomide (Len) and pomalidomide (POM), with strong immune stimulatory activity [42].

The results of the first-in-human phase 1 study of CC-92480 combined with dexamethasone (dex) in patients with RRMM were presented [43]. In a multicenter, dose escalation study, the MTD, RP2D, safety, tolerability, and pharmacokinetics of CC-92480 + dex were tested in heavily pretreated RRMM patients with a median of six prior regimens. Nearly three quarters of the patients were refractory to lenalidomide and pomalidomide (pom). Various doses and schedules of CC-92480 were explored.

MTD was 1.0 mg for both 10/14-day × 2 and 21/28 schedules. At the time of data cut, of the reported 76 patients that were treated, 51 patients discontinued treatment with progressive disease being the most common reason. Common TEAEs (all grade, >10% occurrence) included cytopenias of all three lineages, infections, fatigue, pyrexia, diarrhea, and nausea. Common (>10%) grade 3–4 TEAEs included neutropenia, anemia, and infections. It is of note that no patients discontinued treatment due to treatment-related AEs. The most common dose-limiting toxicity was neutropenia. ORR was 21% (nine very good partial responses (VGPRs); six complete responses (CRs)) for efficacy evaluable population (*n* = 76). Efficacy was dose and schedule dependent; across two 1.0 mg QD schedules (10/14 × 2 and 21/28), 12 of 21 (57%) patients responded (5 ≥VGPR and 5 PR), with responses independent of immunomodulatory drug (IMiD) refractoriness. Eleven patients were treated at the RP2D of 1 mg QD (21/28 day schedule) and the ORR was 54.5% with ≥VGPR of 27.3%.

Discussion: CC-92480 in combination with dexamethasone has shown activity in MM resistant to both first and second-generation IMiDs and seems tolerable. It has shown promising activity in patients with triple-class and pom-refractory myeloma. Its potent tumoricidal activity is evident with the responses seen in extramedullary plasmacytomas in the study. The study is ongoing with a dose-expansion cohort at the RP2D. A phase 1/2 study testing the safety and efficacy of CC-92480 with standard treatments in RRMM is also ongoing. 

#### 3.3.5. BELLINI Study (Abstract 8509)

Venetoclax (Ven) is a selective, potent, oral BCL-2 inhibitor. In the multicenter, randomized, double-blind BELLINI study, patients with RRMM with 1–3 prior lines of therapy and non-refractory to proteasome inhibitors were randomized 2:1 to Ven (800 mg PO) daily or placebo (Pbo) in combination with bortezomib (B) + dexamethasone (d) until progression. Addition of Ven to B and d significantly improved response rates and progression-free survival (PFS) vs. placebo (Pbo) and showed significant efficacy in patients with either t(11;14) or BCL2 high gene expression [44]. Updated results from the BELLINI study were presented at the ASCO scientific symposium this year [45].

The primary endpoint was PFS; key secondary endpoints included ORR and OS. In the Ven arm, the median age of patients was 66 years, 17% had high-risk cytogenetics, 10% had t(11;14), and 78% had BCL2high gene expression. Most common (>20% of patients) TEAEs with Ven were diarrhea, nausea, and constipation. Most common grade 3/4 AEs (>15% of patients) with Ven were neutropenia, thrombocytopenia, anemia, diarrhea and pneumonia. Serious AEs occurred in 54% Ven and 52% Pbo patients. Treatment discontinuation due to AEs were seen in 24% in the Ven arm vs. 12% in the Pbo arm. There were 14 treatment-emergent deaths in the Ven arm and 1 in Pbo.

Discussion: Addition of Ven to Bd significantly improved PFS but resulted in increased deaths compared to placebo. The greatest PFS improvement with Ven was observed in patients with t(11;14) or BCL2high gene expression. In a separate phase 1/2 study reported by Kauffman et al. at the symposium, the combination of Venetoclax/daratumumab/dexamethasone (VDd) with or without bortezomib (B) in RRMM showed increased ORR and VGPR or better in the arm without bortezomib (ORR of 92% and 96% with 79% and 96% VGPR or better in the VDd + B and VDd arms respectively). Also, among patients achieving CR/sCR, significantly more patients in the arm without bortezomib achieved MRD negativity. At the time of reporting the data, median DoR and median PFS have not been reached. No treatment-related deaths were reported in either arm [46].

## 4. Systemic AL Amyloidosis

Patients with amyloidosis have fewer treatment options and worse prognosis compared to patients with multiple myeloma. Two studies that were presented involving treatment of AL amyloidosis are discussed here.

### 4.1. TOURMALINE-AL 1 Trial (Abstract 8546)

In the TOURMALINE-AL 1 trial, the first phase-3 open-label, randomized trial conducted in relapsed/refractory (RR) primary systemic AL amyloidosis (RRAL) with cardiac or renal involvement, Ixazomib-dexamethasone (Ixa-dex) was compared to physician’s choice (PC) by prior proteasome inhibitor (PI) exposure. Data presented at ASH meeting last year showed that although the first primary endpoint of hematologic ORR was not met, all other clinically relevant time-to-event endpoint data favored Ixa-Dex vs. PC [47].

In the symposium this year, subgroup analyses of ORR and outcomes and safety by prior PI exposure were reported [48] (Table 6). In the current study, RRAL patients with 1–2 prior therapies were randomized (1:1) to Ixa-Dex (*n* = 85) or PC (*n* = 83) including lenalidomide-dex, melphalan-dex, cyclophosphamide-dex, or thalidomide-dex. Patients were stratified by cardiac risk stage, relapsed vs. refractory disease, and prior PI exposure. The primary endpoints were hematologic ORR and 2-yr rate of vital organ deterioration or death.

Of the 168 patients enrolled, 90 were PI-naïve and 78 PI-exposed (46 and 39 in the Ixa-Dex arm; 44 and 39 in the PC arm respectively). For time-to-event outcomes, see Table 6. Hazard ratios (HRs) were 0.46–0.85 in favor of Ixa-Dex vs. PC in both PI-naïve and PI-exposed patients.

Discussion: Hematologic ORR was higher with Ixa-dex vs. PC in PI naïve patients (63% vs. 50%, odds ratio [OR] 1.71; 95% confidence interval [CI] 0.74–3.96) but lower in PI exposed patients (41% vs. 51%, [OR] 0.66; 95% CI 0.27–1.62). In terms of clinical outcomes with Ixa-dex vs. PC, the data are discordant, showing an improved two-year time to vital organ deterioration or death in the PI-exposed group but not in the PI-naïve group, and longer time to treatment failure in the PI-naïve group but not in the PI-exposed group. Given the paucity of treatment options for RRAL patients, ixazomib can be considered in both PI-exposed and PI-naïve RRAL patients in the appropriate clinical scenario.

### 4.2. Ixazomib, Cyclophosphamide and Dexamethasone in Newly Diagnosed AL Amyloidosis (Abstract 8065)

Results of a phase 1/2, open-label, multi institutional study to assess the safety and dosage of ixazomib in combination with cyclophosphamide and dexamethasone in newly diagnosed patients with AL amyloidosis were presented [49]. Patients 18 years or older with newly diagnosed, untreated, biopsy-proven AL amyloidosis were eligible. The total accrual goal is 30, with up to 18 in the dose escalation arm (phase 1) and 12 in the expansion arm (phase 2) according to a classical 3 + 3 design. The primary study objective in phase 1 was to establish the MTD and in phase 2 to determine hematologic/organ response rate.

Four dose levels were evaluated in phase 1. The MTD was established at dose level 3 (Ixazomib 4 mg and cyclophosphamide 500 mg). Ixazomib and cyclophosphamide were given orally (PO) on days 1, 8, 15, and dexamethasone 20 mg PO on days 1, 8, 15, 22 of each 28-day cycle. At the time of reporting the data, 16 patients were enrolled to the phase-1 arm and 4 patients to the phase-2 arm. 

The median age of patients was 65 years. Multi-organ involvement was seen in 22% of the patients. Four patients (22%) completed six cycles of therapy and six (33%) remain on study with a median of three cycles completed. Eight of 16 patients (50%) had at least one drug-related AE (any grade), most commonly edema fatigue, dizziness/lightheadedness and lymphopenia. Grade-3/4 AEs were rare with grade-3 lymphopenia, anemia, and hyponatremia occurring in 13%, 6%, and 6% of patients respectively. Of 18 evaluable patients, 7 (39%) achieved ≥VGPR with the median time to best response 2 cycles.

Discussion: The combination of Ixazomib with cyclophosphamide and dexamethasone is an attractive all-oral drug regimen for the treatment of newly diagnosed AL amyloidosis. The lower rate of neuropathy with Ixazomib makes it an alternative to CyBorD regimen for patients who cannot tolerate bortezomib. While it is too early to make comparison, time-to-response appears similar to CyBorD in newly diagnosed AL amyloidosis [50]. Depth of response needs longer follow-up.

## 5. Minimal Residual Disease in Multiple Myeloma

Minimal residual disease (MRD) status after treatment is emerging as one of the most powerful surrogate markers of survival in multiple myeloma. Multiparameter flow cytometry (MFC) and next generation sequencing (NGS) have been two of the most commonly used methods to measure MRD in clinical practice, while mass spectrometry (MS) to detect a monoclonal protein in the peripheral blood has been recently introduced as a potential novel alternative. In spite of a lot of data available on the performance of each method, there is little data on the concordance between them.

### FORTE Trial (Abstract 8533)

In the FORTE trial, patients ≤65 years with NDMM were randomized in a 1:1:1 fashion to carfilzomib, lenalidomide, dexamethasone (KRd) induction–ASCT–KRd consolidation (KRd_ASCT); 12 KRd cycles (KRd12); carfilzomib, cyclophosphamide, dexamethasone (KCd) induction–ASCT–KCd consolidation (KCd_ASCT). Patients then underwent a second randomization to maintenance with either lenalidomide alone until progression/intolerance or carfilzomib for two years plus lenalidomide until progression/intolerance. At the ASCO symposium this year, data on MRD negativity and the concordance between NGS and MFC were reported [51]. MRD was assessed by MFC (sensitivity 10^−5^) in patients with ≥ VGPR and by both MFC and NGS in patients who were in CR or better before maintenance. One-year sustained MRD negativity by MFC and NGS was also analyzed in patients with at least two samples available at least one year apart. MFC and NGS data were available in 184/233 (79%) CR patients (66 KRd_ASCT, 67 KRd12, and 51 KCd_ASCT). For the KRD-ASCT, KRD12, and KCd_ASCT arms, the rates of MRD negativity by ≥10^−5^ MFC were 83%, 79%, and 69%; by 10^−5^ NGS were 76%, 69%, 70%; and by 10^−6^ NGS were 34%, 23%, and 27% respectively. 

Discussion: In evaluable patients, one-year sustained 10^−5^ MRD negativity by MFC and NGS was superimposable (83%), to suggest concordance between MFC and NGS, particularly when the same sensitivity was reached. However, looking at MRD status pre-maintenance in the three groups a discordance between 1–10 percent was noted at 10^−5^ sensitivity, with MFC technique resulting in more cases that were positive. Given the emerging role of MRD status in the management of MM, the significance of this discrepancy needs to be further addressed in clinical trials with longer follow-up.

## Figures and Tables

**Table 1:** Treatment, efficacy, and safety data from the ENDURANCE (E1A11) trial.

	VRd	KRd
Median induction duration (mos)	6.5	8.9
Reason off study		
Disease progression	6%	4%
Adverse events	17.3%	9.9%
Alternative therapy	18%	14%
Patient withdrawal	7%	4%
Response		
≥PR	84%	87%
≥VGPR	65%	73%
≥CR	15%	16.5%
Toxicity grade ≥3		
Non-hematological	41.4%	48.2%
Composite cardiac/pulmonary/renal	5%	16%
Peripheral neuropathy	8%	1%

mos, months; PR, partial response; VGPR, very good partial response; CR, complete response

**Table 2:** Efficacy and safety data from the TOURMALINE-MM4 trial.

		Ixazomib	Placebo	HR (95% CI)
	Median PFS, months	17.4	9.4	0.659 (0.542–0.801) *p* < 0.001
	≥VGPR post induction (%)	25.6	12.9	0.586 (0.449–0.765) *p* < 0.001
	Pre-induction ISS stage III	16.6	7.8	0.695 (0.499–0.967) *p* = 0.03
	Age ≥75 yrs	16.7	10.6	0.738 (0.537–1.014) *p* = 0.06
Safety, %	Any TEAE	91	82	
	Grade ≥3 TEAE	37	23	
	Serious TEAE	22	17	
	Discontinuation due to TEAE	13	8	
	On-study death	2.6	2.2	

PFS, progression-free survival; VGPR, very good partial response; ISS, International staging system; TEAE, Treatment-emergent adverse event.

**Table 3:** Efficacy and safety data from the KarMMa study.

Dose × 10^6^ CAR + T cells	150 (*n* = 4)	300 (*n* = 70)	450 (*n* = 54)	Total (*n* = 128)
ORR, *n* (%)	2 (50)	48 (69)	44 (82)	93.4 (73)
CR/sCR, *n* (%)	1 (25)	20 (29)	21 (39)	42 (33)
Median DoR *, months	^†^	9.9	11.3	10.7
Median PFS *, months	2.8	5.8	12.1	8.8
CRS overall/Gr ≥ 3, *n* (%)	2 (50)/0	53 (76)/4 (6)	52 (96)/3 (6)	107 (84)/7 (5)
Median onset/duration, days	7/5	2/4	1/7	1/5
NT overall/Gr ≥ 3, *n* (%)	0/0	12 (17)/1 (1)	11 (20)/3 (6)	23 (18)/4 (3)
Median onset/duration, days	NA	3/3	2/5	2/3

ORR, overall response rate; CR, complete response; sCR, stringent CR; DoR, duration of response; PFS, progression-free survival; CRS, cytokine release syndrome; NT, investigator identified neurotoxicity. * Kaplan-Meier estimate. ^†^ Not reported due to small *n*.

**Table 4:** Efficacy and safety data from the EVOLVE study.

*n* (%)	300 × 10^6^ CAR + T cells	450 × 10^6^ CAR + T cells	600 × 10^6^ CAR + T cells	All DLs
Efficacy	*n* = 19	*n* = 19	*n* = 24	*n* = 62
ORR (sCR + CR + VGPR + PR)	18 (95)	17 (89)	22 (92)	57 (92)
sCR + CR	7 (37)	8 (42)	7 (29)	22 (36)
VGPR	7 (37)	5 (26)	8 (33)	20 (32)
PR	4 (21)	4 (21)	7 (29)	15 (24)
Median F/U, months	9.5	8.8	2.3	6.9
PFS	9.3	NR	NR	
Safety	*n* = 19	*n* = 19	*n* = 24	*n* = 62
CRS grade ≥3	0	1 (5)	1 (4)	2 (3)
NE grade ≥3	1 (5)	1 (5)	0	2 (3)
MAS	0	2 (11)	1 (4)	3 (5)

ORR, overall response rate; sCR, stringent complete response; PR, partial response; VGPR, very good PR; F/U, follow-up; PFS, progression-free survival; NR, not reached; CRS, cytokine release syndrome; NE, neurological events; MAS, macrophage activation syndrome.

**Table 5:** Comparison of the three chimeric antigen receptor (CAR-) T cell products.

	CARTITUDE: JNJ-4528: (*n* = 29)	KarMMa: Idecabtagene Vicleucel: (*n* = 128)	EVOLVE: Orvacabtagene Autoleucel: (*n* = 62)
Structure	2 BCMA single chain antibodies	Human BCMA, 4–1 BB, CD3z	Modified spacer
Age	60 (50–75)	61 (33–78)	61 (33–77)
High-risk cytogenetics %	27	35	41
Median prior lines of therapy	5 (3–18)	6 (3–16)	6 (3–18)
Neutropenia/thrombocytopenia, ≥G3, %	100/69	89/52	90/47
CRS: all, ≥G3, %	93, 7	84, 6	89, 3
ICANS: all, ≥G3, %	10, 3	17, 3	13, 3
Infections: all, ≥G3, %	NR, 19	69, NR	40, 13
ORR/ ≥CR, %	100/86	92/33	73/36
MRD neg ≥10^−5^, % (of evaluable)	81	94	84
PFS/DoR, months	NR **	8.8/10.7	NR *

BCMA, B-cell maturation antigen; CRS, cytokine release syndrome; ICANS, Immune effector cell-associated neurotoxicity; ORR, overall response rate; CR, complete response; MRD, minimal residual disease; PFS, progression-free survival; DoR, Duration of response; NR, not reported; * 9 month PFS = 86%; ** 300 × 10 cell dose cohort (lowest) = PFS 9.3 months, other median follow-up = 8.8 and 2.3 months

**Table 6:** Time-to-event outcomes for patients with AL amyloidosis treated with Ixa-Dex vs. physician’s choice.

	PI Naïve			PI Exposed		
Median, mos	Ixa-Dex	Physician’s choice	HR (95% CI)	Ixa-Dex	Physician’s choice	HR (95% CI)
Time to vital organ deterioration or death	44.9	28.0	0.53 (0.24–1.18); *p* = 0.112	27.0	26.1	0.52 (0.27–1.01); *p* = 0.050
Composite progression-free survival	30.4	9.8	0.56 (0.31–1.02); *p* = 0.054	7.0	5.5	0.77 (0.46–1.28); *p* = 0.309
Time to treatment failure	16.2	5.3	0.46 (0.27–0.79); *p* = 0.004	7.0	5.2	0.76 (0.47–1.23); *p* = 0.262
Time to subsequent therapy	61.4	16.3	0.57 (0.29–1.09); *p* = 0.084	15.7	12.1	0.66 (0.37–1.18); *p* = 0.156
Overall survival	Not reached	71.1	0.81 (0.37–1.80); *p* = 0.610	40.9	32.4	0.85 (0.46–1.60); *p* = 0.616

PI, proteasome inhibitor; mos, months; HR, hazard ratio; CI, confidence interval.

## References

[1] Moreau P., Attal M., Hulin C., Arnulf B., Belhadj K., Benboubker L., Béné M.C., Broijl A., Caillon H., Caillot D. (2019). Bortezomib, thalidomide, and dexamethasone with or without daratumumab before and after autologous stem-cell transplantation for newly diagnosed multiple myeloma (CASSIOPEIA): A randomised, open-label, phase 3 study. Lancet.

[2] Touzeau C., Moreau P., Perrot A., Hulin C., Dib M., Tiab M., Caillot D., Facon T., Leleu X., Van De Donk N.W. (2020). Daratumumab + bortezomib, thalidomide, and dexamethasone (D-VTd) in transplant-eligible newly diagnosed multiple myeloma (TE NDMM): Baseline SLiM-CRAB based subgroup analysis of CASSIOPEIA. J. Clin. Oncol..

[3] Rajkumar S.V., Dimopoulos M.A., Palumbo A., Blade J., Merlini G., Mateos M.V., Kumar S., Hillengass J., Kastritis E., Richardson P. (2014). International Myeloma Working Group updated criteria for the diagnosis of multiple myeloma. Lancet Oncol..

[4] Jacobus S.J., Kumar S., Uno H., Van Wier S.A., Ahmann G.J., Henderson K.J., Callander N., Williams M.E., Siegel D.S., Greipp P.R. (2011). Impact of high-risk classification by FISH: An Eastern Cooperative Oncology Group (ECOG) study E4A03. Br. J. Haematol..

[5] Weisel K., Asemissen A.M., Besemer B., Haenel M., Blau I.W., Goerner M., Ko Y.-D., Dürig J., Staib P., Mann C. (2020). Depth of response to isatuximab, carfilzomib, lenalidomide, and dexamethasone (Isa-KRd) in front-line treatment of high-risk multiple myeloma: Interim analysis of the GMMG-CONCEPT trial. J. Clin. Oncol..

[6] Voorhees P.M., Kaufman J.L., Laubach J.P., Sborov D.W., Reeves B., Rodriguez C., Chari A., Silbermann R.W., Costa L.J., Anderson L.D. (2019). Depth of Response to Daratumumab (DARA), Lenalidomide, Bortezomib, and Dexamethasone (RVd) Improves over Time in Patients (pts) with Transplant-Eligible Newly Diagnosed Multiple Myeloma (NDMM): Griffin Study Update. Blood.

[7] Costa L.J., Chhabra S., Godby K.N., Medvedova E., Cornell R.F., Hall A.C., Silbermann R.W., Innis-Shelton R., Dhakal B., DeIdiaquez D. (2019). Daratumumab, Carfilzomib, Lenalidomide and Dexamethasone (Dara-KRd) Induction, Autologous Transplantation and Post-Transplant, Response-Adapted, Measurable Residual Disease (MRD)-Based Dara-Krd Consolidation in Patients with Newly Diagnosed Multiple Myeloma (NDMM). Blood.

[8] Usmani S.Z., Ailawadhi S., Sexton R., Hoering A., Lipe B., Hita S., Durie B.G., Zonder J.A., Dhodapkar M.V., Callander N.S. (2020). Primary analysis of the randomized phase II trial of bortezomib, lenalidomide, dexamthasone with/without elotuzumab for newly diagnosed, high-risk multiple myeloma (SWOG-1211). J. Clin. Oncol..

[9] Dimopoulos M.A., Dytfeld D., Grosicki S., Moreau P., Takezako N., Hori M., Leleu X., Leblanc R., Suzuki K., Raab M.S. (2018). Elotuzumab plus Pomalidomide and Dexamethasone for Multiple Myeloma. N. Engl. J. Med..

[10] Lonial S., Dimopoulos M.A., Palumbo A., White D., Grosicki S., Spicka I., Walter-Croneck A., Moreau P., Mateos M.V., Magen H. (2015). Elotuzumab Therapy for Relapsed or Refractory Multiple Myeloma. New Engl. J. Med..

[11] Kumar S., Jacobus S.J., Cohen A.D., Weiss M., Callander N.S., Singh A.A., Parker T.L., Menter A.R., Yang X., Parsons B.M. (2020). Carfilzomib, lenalidomide, and dexamethasone (KRd) versus bortezomib, lenalidomide, and dexamethasone (VRd) for initial therapy of newly diagnosed multiple myeloma (NDMM): Results of ENDURANCE (E1A11) phase III trial. J. Clin. Oncol..

[12] Dimopoulos M.A., Goldschmidt H., Niesvizky R., Joshua D., Chng W.-J., Oriol A., Orlowski R.Z., Ludwig H., Facon T., Hajek R. (2017). Carfilzomib or bortezomib in relapsed or refractory multiple myeloma (ENDEAVOR): An interim overall survival analysis of an open-label, randomised, phase 3 trial. Lancet Oncol..

[13] Durie B.G.M., Hoering A., Abidi M.H., Rajkumar S.V., Epstein J., Kahanic S.P., Thakuri M., Reu F., Reynolds C.M., Sexton R. (2017). Bortezomib with lenalidomide and dexamethasone versus lenalidomide and dexamethasone alone in patients with newly diagnosed myeloma without intent for immediate autologous stem-cell transplant (SWOG S0777): A randomised, open-label, phase 3 trial. Lancet.

[14] Stadtmauer E.A., Pasquini M.C., Blackwell B., Hari P., Bashey A., Devine S., Efebera Y., Ganguly S., Gasparetto C., Geller N. (2019). Autologous Transplantation, Consolidation, and Maintenance Therapy in Multiple Myeloma: Results of the BMT CTN 0702 Trial. J. Clin. Oncol..

[15] Parameswaran H., Pasquini C.M., Stadtmauer A.E., Fraser R., Fei M., Devine M.S., Efebera Y.A., Geller N., Horowitz M.M., Koreth J. (2020). Long-term follow-up of BMT CTN 0702 (STaMINA) of post autologous hematopoietic cell transplantation (autoHCT) strategies in the upfront treatment of multiple myeloma (MM). J. Clin. Oncol..

[16] Dimopoulos M.A., Spicka I., Quach H., Oriol A., Hajek R., Garg M., Beksac M., Bringhen S., Katodritou E., Chng W.J. (2020). Ixazomib vs placebo maintenance for newly diagnosed multiple myeloma (NDMM) patients not undergoing autologous stem cell transplant (ASCT): The phase III TOURMALINE-MM4 trial. J. Clin. Oncol..

[17] Jackson G.H., Davies F.E., Pawlyn C., Cairns D.A., Striha A., Collett C., Hockaday A., Jones J.R., Kishore B., Garg M. (2019). Lenalidomide maintenance versus observation for patients with newly diagnosed multiple myeloma (Myeloma XI): A multicentre, open-label, randomised, phase 3 trial. Lancet Oncol..

[18] Maude S.L., Laetsch T.W., Buechner J., Rives S., Boyer M., Bittencourt H., Bader P., Verneris M.R., Stefanski H.E., Myers G.D. (2018). Tisagenlecleucel in Children and Young Adults with B-Cell Lymphoblastic Leukemia. N. Engl. J. Med..

[19] Schuster S.J., Svoboda J., Chong E.A., Nasta S.D., Mato A., Anak Ö., Brogdon J.L., Pruteanu-Malinici I., Bhoj V., Landsburg D. (2017). Chimeric Antigen Receptor T Cells in Refractory B-Cell Lymphomas. N. Engl. J. Med..

[20] Wang B.-Y., Zhao W.-H., Liu J., Chen Y.-X., Cao X.-M., Yang Y., Zhang Y.-L., Wang F.-X., Zhang P.-Y., Lei B. (2019). Long-Term Follow-up of a Phase 1, First-in-Human Open-Label Study of LCAR-B38M, a Structurally Differentiated Chimeric Antigen Receptor T (CAR-T) Cell Therapy Targeting B-Cell Maturation Antigen (BCMA), in Patients (pts) with Relapsed/Refractory Multiple Myeloma (RRMM). Blood.

[21] Madduri D., Usmani S.Z., Jagannath S., Singh I., Zudaire E., Yeh T.-M., Allred A.J., Banerjee A., Goldberg J.D., Schecter J.M. (2019). Results from CARTITUDE-1: A Phase 1b/2 Study of JNJ-4528, a CAR-T Cell Therapy Directed Against B-Cell Maturation Antigen (BCMA), in Patients with Relapsed and/or Refractory Multiple Myeloma (R/R MM). Blood.

[22] Berdeja J.G., Madduri D., Usmani S.Z., Singh I., Zudaire E., Yeh T.-M., Allred A.J., Olyslager Y., Banerjee A., Goldberg J.D. (2020). Update of CARTITUDE-1: A phase Ib/II study of JNJ-4528, a B-cell maturation antigen (BCMA)-directed CAR-T-cell therapy, in relapsed/refractory multiple myeloma. J. Clin. Oncol..

[23] Munshi N.C., Anderson J.L.D., Shah N., Jagannath S., Berdeja J.G., Lonial S., Raje N.S., Siegel D.S.D., Lin Y., Oriol A. (2020). Idecabtagene vicleucel (ide-cel; bb2121), a BCMA-targeted CAR T-cell therapy, in patients with relapsed and refractory multiple myeloma (RRMM): Initial KarMMa results. J. Clin. Oncol..

[24] Mailankody S., Jakubowiak A.J., Htut M., Costa L.J., Lee K., Ganguly S., Kaufman J.L., Siegel D.S.D., Bensinger W., Cota M. (2020). Orvacabtagene autoleucel (orva-cel), a B-cell maturation antigen (BCMA)-directed CAR T cell therapy for patients (pts) with relapsed/refractory multiple myeloma (RRMM): Update of the phase 1/2 EVOLVE study (NCT03430011). J. Clin. Oncol..

[25] Kumar S., Paiva B., Anderson K.C., Durie B., Landgren O., Moreau P., Munshi N.V., Lonial S., Bladé J., Mateos M.-V. (2016). International Myeloma Working Group consensus criteria for response and minimal residual disease assessment in multiple myeloma. Lancet Oncol..

[26] Tai Y.-T., Mayes P.A., Acharya C., Zhong M.Y., Cea M., Cagnetta A., Craigen J., Yates J.R.W., Gliddon L., Fieles W. (2014). Novel anti–B-cell maturation antigen antibody-drug conjugate (GSK2857916) selectively induces killing of multiple myeloma. Blood.

[27] Tai Y.T., Anderson K.C. (2015). Targeting B-cell maturation antigen in multiple myeloma. Immunotherapy.

[28] Nooka A.K., Stockerl-Goldstein K., Quach H., Forbes A., Mateos M.-V., Khot A., Tan A., Abonour R., Chopra B., Rogers R. (2020). DREAMM-6: Safety and tolerability of belantamab mafodotin in combination with bortezomib/dexamethasone in relapsed/refractory multiple myeloma (RRMM). J. Clin. Oncol..

[29] Palumbo A., Chanan-Khan A., Weisel K., Nooka A.K., Masszi T., Beksac M., Spicka I., Hungria V., Munder M., Mateos M.V. (2016). Daratumumab, Bortezomib, and Dexamethasone for Multiple Myeloma. N. Engl. J. Med..

[30] San-Miguel J.F., Hungria V.T., Yoon S.S., Beksac M., Dimopoulos M.A., Elghandour A., Jedrzejczak W.W., Günther A., Nakorn T.N., Siritanaratkul N. (2014). Panobinostat Plus Bortezomib and Dexamethasone versus Placebo Plus Bortezomib and Dexamethasone in Patients With Relapsed or Relapsed and Refractory Multiple Myeloma: A Multicenter, Randomized, Double-Blind Phase 3 Trial. Lancet Oncol..

[31] Richardson P.G., Oriol A., Beksac M., Liberati A.M., Galli M., Schjesvold F., Lindsay J., Weisel K., White D., Facon T. (2019). Pomalidomide, bortezomib, and dexamethasone for patients with relapsed or refractory multiple myeloma previously treated with lenalidomide (OPTIMISMM): A randomised, open-label, phase 3 trial. Lancet Oncol..

[32] Chari A., Vogl D.T., Gavriatopoulou M., Nooka A.K., Yee A.J., Huff C.A., Moreau P., Dingli D., Cole C., Lonial S. (2019). Oral Selinexor–Dexamethasone for Triple-Class Refractory Multiple Myeloma. N. Engl. J. Med..

[33] Dimopoulos M.A., Delimpasi S., Simonova M., Spicka I., Pour L., Kryachok I., Gavriatopoulou M., Pylypenko H., Auner H.W., Leleu X. (2020). Weekly selinexor, bortezomib, and dexamethasone (SVd) versus twice weekly bortezomib and dexamethasone (VD) in patients with multiple myeloma (MM) after one to three prior therapies: Initial results of the phase III BOSTON study. J. Clin. Oncol..

[34] Bahlis N.J., Sutherland H., White D., Sebag M., Lentzsch S., Kotb R., Venner C.P., Gasparetto C., Del Col A., Neri P. (2018). Selinexor plus low-dose bortezomib and dexamethasone for patients with relapsed or refractory multiple myeloma. Blood.

[35] Usmani S.Z., Quach H., Mateos M.V., Landgren O., Leleu X., Siegel D.S., Weisel K., Yang H., Klippel Z.K., Zahlten-Kumeli A. (2019). Carfilzomib, Dexamethasone, and Daratumumab Versus Carfilzomib and Dexamethasone for the Treatment of Patients with Relapsed or Refractory Multiple Myeloma (RRMM): Primary Analysis Results from the Randomized, Open-Label, Phase 3 Study Candor (NCT03158688). Blood.

[36] Gasparetto C., Lipe B., Tuchman S., Callander N.S., Lentzsch S., Baljevic M., Rossi A.C., Bahlis N.J., White D., Chen C. (2020). Once weekly selinexor, carfilzomib, and dexamethasone (SKd) in patients with relapsed/refractory multiple myeloma (MM). J. Clin. Oncol..

[37] Fedyk E.R., Berg D., Smithson G., Estevam J., McLean L., Allikmets K., Palumbo A. (2018). A Single Administration of the Cytolytic CD38 Antibody TAK-079 to Healthy Subjects: Tolerability, Pharmacokinetics and Pharmacodynamics. Blood.

[38] Krishnan A.Y., Patel K.K., Hari P., Jagannath S., Niesvizky R., Silbermann R.W., Berg D.T., Li Q., Allikmets K., Stockerl-Goldstein K. (2020). A phase Ib study of TAK-079, an investigational anti-CD38 monoclonal antibody (mAb) in patients with relapsed/refractory multiple myeloma (RRMM): Preliminary results. J. Clin. Oncol..

[39] Lonial S., Weiss B.M., Usmani S.Z., Singhal S., Chari A., Bahlis N.J., Belch A., Krishnan A., Vescio R.A., Mateos M.V. (2016). Daratumumab monotherapy in patients with treatment-refractory multiple myeloma (SIRIUS): An open-label, randomised, phase 2 trial. Lancet.

[40] Mateos M.V., Nahi H., Legiec W., Grosicki S., Vorobyev V., Spicka I., Hungria V., Korenkova S., Bahlis N., Flogegard M. (2020). Subcutaneous versus intravenous daratumumab in patients with relapsed or refractory multiple myeloma (COLUMBA): A multicentre, open-label, non-inferiority, randomised, phase 3 trial. Lancet Haematol..

[41] Dimopoulos M., Bringhen S., Anttila P., Capra M., Cavo M., Cole C.E., Gasparetto C.J., Hungria V.T.M., Jenner M., Vorobyev V. (2018). Results from a Phase II Study of Isatuximab As a Single Agent and in Combination with Dexamethasone in Patients with Relapsed/Refractory Multiple Myeloma. Blood.

[42] Lopez-Girona A., Havens C.G., Lu G., Rychak E., Mendy D., Gaffney B., Surka C., Lu C.-C., Matyskiela M., Khambatta G. (2019). CC-92480 Is a Novel Cereblon E3 Ligase Modulator with Enhanced Tumoricidal and Immunomodulatory Activity Against Sensitive and Resistant Multiple Myeloma Cells. Blood.

[43] Richardson P.G., Vangsted A.J., Ramasamy K., Trudel S., Martínez J., Mateos M.-V., Otero P.R., Lonial S., Popat R., Oriol A. (2020). First-in-human phase I study of the novel CELMoD agent CC-92480 combined with dexamethasone (DEX) in patients (pts) with relapsed/refractory multiple myeloma (RRMM). J. Clin. Oncol..

[44] Moreau P., Harrison M.S., Cavo M., De La Rubia J., Popat R., Gasparetto C., Hungria V.T., Salwender H., Suzuki K., Kim I. (2019). Updated Analysis of Bellini, a Phase 3 Study of Venetoclax or Placebo in Combination with Bortezomib and Dexamethasone in Patients with Relapsed/Refractory Multiple Myeloma. Blood.

[45] Kumar S., Harrison S.J., Cavo M., De La Rubia J., Popat R., Gasparetto C., Hungria V., Salwender H., Suzuki K., Kim I. (2020). Updated results from BELLINI, a phase III study of venetoclax or placebo in combination with bortezomib and dexamethasone in relapsed/refractory multiple myeloma. J. Clin. Oncol..

[46] Kaufman J.L., Baz R.C., Harrison S.J., Quach H., Ho S.-J., Vangsted A.J., Moreau P., Gibbs S.D., Salem A.H., Coppola S. (2020). Updated analysis of a phase I/II study of venetoclax in combination with daratumumab and dexamethasone, +/- bortezomib, in patients with relapsed/refractory multiple myeloma. J. Clin. Oncol..

[47] Dispenzieri A., Kastritis E., Wechalekar A.D., Schönland S.O., Kim K., Sanchorawala V., Landau H.J., Kwok F., Suzuki K., Comenzo R.L. (2019). Primary Results from the Phase 3 Tourmaline-AL1 Trial of Ixazomib-Dexamethasone Versus Physician’s Choice of Therapy in Patients (Pts) with Relapsed/Refractory Primary Systemic AL Amyloidosis (RRAL). Blood.

[48] Kastritis E., Dispenzieri A., Wechalekar A.D., Schönland S.O., Kim K., Sanchorawala V., Landau H.J., Kwok F., Suzuki K., Comenzo R. (2020). Ixazomib-dexamethasone (Ixa-Dex) vs physician’s choice (PC) in relapsed/refractory (RR) primary systemic AL amyloidosis (AL) patients (pts) by prior proteasome inhibitor (PI) exposure in the phase III TOURMALINE-AL1 trial. J. Clin. Oncol..

[49] Osman K. (2020). A phase I/II study to assess safety and dose of ixazomib in combination with cyclophosphamide and dexamethasone in newly diagnosed patients with light chain (AL) amyloidosis. J. Clin. Oncol..

[50] Mikhael J.R., Schuster S.R., Jimenez-Zepeda V.H., Bello N., Spong J., Reeder C.B., Stewart A.K., Bergsagel P.L., Fonseca R. (2012). Cyclophosphamide-bortezomib-dexamethasone (CyBorD) produces rapid and complete hematologic response in patients with AL amyloidosis. Blood.

[51] Oliva S., Genuardi E., Belotti A., Frascione P.M.M., Galli M., Capra A., Offidani M., Vozella F., Zambello R., Auclair D. (2020). Multiparameter flow cytometry (MFC) and next generation sequencing (NGS) for minimal residual disease (MRD) evaluation: Results of the FORTE trial in newly diagnosed multiple myeloma (MM). J. Clin. Oncol..

